# Possible Gastroenterological Causes of FUO (Fever of Unknown Origin)

**DOI:** 10.3390/jcm15114350

**Published:** 2026-06-04

**Authors:** Oliwia Cichy, Aleksandra Wojno, Agata Wojno, Anna Karwowska, Olgierd Dróżdż, Maciej Rabczyński, Katarzyna Madziarska

**Affiliations:** 1 Student Scientific Association, Clinical Department of Diabetology, Hypertension and Internal Diseases, Institute of Internal Diseases, Wroclaw Medical University, 50-556 Wroclaw, Poland; aleksandra.wojno@student.umw.edu.pl (A.W.); agata.wojno@student.umw.edu.pl (A.W.); anna.karwowska@student.umw.edu.pl (A.K.); 2 Clinical Department of Diabetology, Hypertension and Internal Diseases, Institute of Internal Diseases, Wroclaw Medical University, 50-556 Wroclaw, Poland; olgierd.drozdz@usk.wroc.pl (O.D.); maciej.rabczynski@umw.edu.pl (M.R.); katarzyna.madziarska@umw.edu.pl (K.M.)

**Keywords:** fever of unknown origin, gastrointestinal diseases, inflammatory bowel disease, gut-associated lymphoid tissue, diagnostic algorithms, intra-abdominal infections, gastrointestinal malignancies

## Abstract

Fever of unknown origin (FUO) remains a persistent diagnostic challenge in clinical medicine despite significant advances in laboratory testing and imaging techniques. The definition of FUO has evolved since the original criteria proposed in 1961 and currently refers to persistent fever exceeding approximately 38.2–38.3 °C without a definitive diagnosis after an adequate diagnostic evaluation. Gastrointestinal diseases represent an important but often underrecognized group of conditions associated with FUO. The aim of this review is to synthesize current evidence on the gastroenterological causes of FUO, with particular emphasis on pathophysiological mechanisms, diagnostic strategies, and therapeutic management. The analysis highlights the role of inflammatory, infectious, and neoplastic gastrointestinal disorders in the etiology of prolonged fever. Key mechanisms involve systemic inflammatory responses mediated by cytokines such as interleukin-1, interleukin-6, and tumor necrosis factor, as well as immune processes associated with the gut-associated lymphoid tissue (GALT) and interactions between intestinal microbiota and host immunity. Among the most frequently reported gastroenterological causes of FUO are inflammatory bowel diseases, intra-abdominal infections and abscesses, hepatobiliary disorders, pancreatitis, and gastrointestinal malignancies. Diagnostic evaluation requires a stepwise approach integrating laboratory testing, microbiological studies, imaging modalities, and endoscopic procedures, with advanced techniques such as computed tomography and fluorodeoxyglucose positron emission tomography improving detection of occult inflammatory or neoplastic processes. Therapeutic management is primarily guided by the identification of the underlying cause, while empirical treatment should be carefully considered to avoid masking diagnostic clues. A better understanding of the gastrointestinal mechanisms underlying FUO and the development of more efficient diagnostic algorithms may improve clinical outcomes and reduce the number of undiagnosed cases.

## 1. Introduction

The concept of fever of unknown origin (FUO) has remained one of the most challenging diagnostic problems in medicine for more than six decades. Its definition has undergone multiple revisions. The classic criteria proposed by Petersdorf and Beeson (1961) included a fever ≥ 38.3 °C lasting for more than three weeks and the absence of a definitive diagnosis despite one week of hospitalization [1]. Durack and Street (1991) adapted the definition to contemporary clinical practice by recognizing the lack of diagnosis after three days of inpatient evaluation or three outpatient visits, and by introducing four categories of FUO: classical, nosocomial, neutropenic, and HIV-associated FUO [2,3]. Subsequently, Mulders-Manders et al. [4] suggested standardizing a minimal set of mandatory investigations to reduce diagnostic misclassification. The classical definition requires documented, persistent fever and adequate inpatient or outpatient diagnostic assessment, including laboratory and imaging studies [5]. Currently, a modified definition is widely used, involving a temperature > 38.2 °C in at least three measurements and the absence of a diagnosis after one week of inpatient diagnostic evaluation [6].

Cohort studies indicate that FUO accounts for up to 3% of hospitalizations, with its etiology varying by region, level of socioeconomic development, and study criteria [3,7]. Despite the availability of modern imaging modalities and expanded diagnostic protocols, as many as 50% of patients may remain without a definitive diagnosis [8]. Analyses based on hospital registry data demonstrate substantial variability in the quality of clinical coding, which hampers precise epidemiological estimates [9]. Additionally, a subset of FUO cases presents without elevated inflammatory markers, particularly CRP, which reduces the diagnostic utility of certain imaging techniques, including PET-CT [10].

The standard epidemiological classification comprises five etiological groups: infectious diseases (INF), non-infectious inflammatory diseases (NIID), malignancies (MAL), miscellaneous causes (MISC), and undiagnosed cases (UI) [7]. Until the late twentieth century, INF constituted the predominant cause of FUO; however, in recent years, a systematic increase in the proportion of NIID and UI has been observed in highly developed countries [7,11,12,13,14,15]. Before initiating a targeted diagnostic workup for FUO, it is essential to confirm the actual presence of fever and to distinguish it from a short-lived fever without an identifiable source, which may merely represent a transient condition [15]. The indiscriminate use of an FUO diagnosis leads to substantial heterogeneity within studied cohorts. It has been shown that the accuracy of coding this diagnosis is markedly higher in infectious disease centers than in other internal medicine departments, which directly affects the reliability of epidemiological analyses and comparisons across studies [9]. It should also be emphasized that the distribution of FUO etiologies changes with age: specifically, the proportion of infections decreases after infancy, whereas the relative importance of inflammatory diseases, malignancies, and other less common diagnoses increases [15,16].

Processes originating within the gastrointestinal system constitute an important component of the etiology of FUO. The literature identifies numerous pathological foci located within the abdominal cavity that may generate prolonged fever, such as intra-abdominal abscesses [13]; malignancies [7]; Crohn’s disease [11]; ulcerative colitis [17]; inflammatory conditions of the liver, biliary tract, and pancreas [11,15,18]; and bacterial and parasitic infections [16,17].

## 2. Pathophysiology of Fever and Immunological Mechanisms in the Gastrointestinal Tract

Fever represents a generalized systemic response to inflammation, in which the activation of innate immune pathways leading to the release of pro-inflammatory cytokines plays a central role [7,12]. In particular, interleukin-1 (IL-1) and interleukin-6 (IL-6) induce the production of prostaglandin E by cerebral endothelial cells, constituting the principal regulatory mechanism responsible for elevating core body temperature [19,20]. Interferons and tumor necrosis factor (TNF) are also of major significance, as they can induce fever either through direct effects on thermoregulatory centers or by enhancing the synthesis of other mediators, including IL-1 [19]. Moreover, the inflammatory response observed in FUO encompasses a broad spectrum of immunological mechanisms, such as leukocytosis, elevated C-reactive protein concentrations, and increased lactate dehydrogenase activity [5,21,22]. It has also been proposed that certain chromosomal deletions and duplications may involve genes involved in the regulation of immune responses, such as TNFAIP3, whose loss may predispose individuals to uncontrolled activation of inflammatory pathways [20]. In the pathophysiology of FUO, it is also important to distinguish between infectious inflammation and sterile autoinflammatory inflammation. In autoinflammatory diseases, disturbances of innate immunity predominate, most commonly those dependent on interleukin-1. However, circulating IL-1 concentrations do not necessarily reflect the activity of the disease process [19]. This may be explained by the fact that the most bioactive form, IL-1β, can remain bound to extracellular DNA traps released by activated neutrophils during NETosis (neutrophil extracellular trap formation), thereby promoting the persistence of the inflammatory response without a straightforward correlation with routine measurements of this cytokine [7].

GALT in the immunological response of the gastrointestinal tract, together with the interactions between the gut microbiota and inflammatory regulation, forms an integrated system that underpins mucosal immunity. The gut-associated lymphoid tissue (GALT) constitutes the principal immunological component of the gastrointestinal tract, accounting for a substantial proportion of the body’s immune activity and remaining in a state of continuous activation under the influence of the intestinal microbiota [23]. It encompasses both organized structures, such as Peyer’s patches and M cells, as well as dispersed lymphocyte populations located within the epithelium and the lamina propria [24,25]. GALT is responsible for antigen sampling, immunological presentation, and the initiation of specific IgA antibody production [24]. Secretory IgA, in turn, limits pathogen adherence to the epithelium, neutralizes toxins, and contributes to maintaining homeostasis between the host and the commensal microbiota through modulation of microbial colonization [25].

It should be emphasized that the gut microbiota constitutes one of the principal regulators of the immune response, acting both through metabolic influence and by modulating the redox state within the intestinal epithelium [25]. The impact of the microbiota on the inflammatory response arises from direct interactions between microbial components and Toll-like receptor (TLR) proteins, as well as from more complex mechanisms involving lymphocyte differentiation, regulation of the intestinal barrier, and modulation of the composition of immune cell populations [23]. Moreover, intestinal dysbiosis results in hyperactivation of GALT; an enhanced Th1 and Th17 response; increased production of IL-12, IL-6, and TNF-α; and further activation of antigen-presenting cells, all of which directly contribute to the development of febrile states [23,26,27,28]. Additionally, it should be taken into account that the role of the gastrointestinal tract in the pathogenesis of febrile states also includes the function of the mucosal barrier, the disruption of which may promote microbial translocation and intensify the systemic inflammatory response. Observations in hematologic patients have demonstrated an association between mucositis, disruption of the intestinal microbiome, and infectious complications, indicating that impairment of gastrointestinal barrier integrity may represent one of the mechanisms linking disturbances in mucosal homeostasis to clinically overt fever [29].

**Figure 1:**
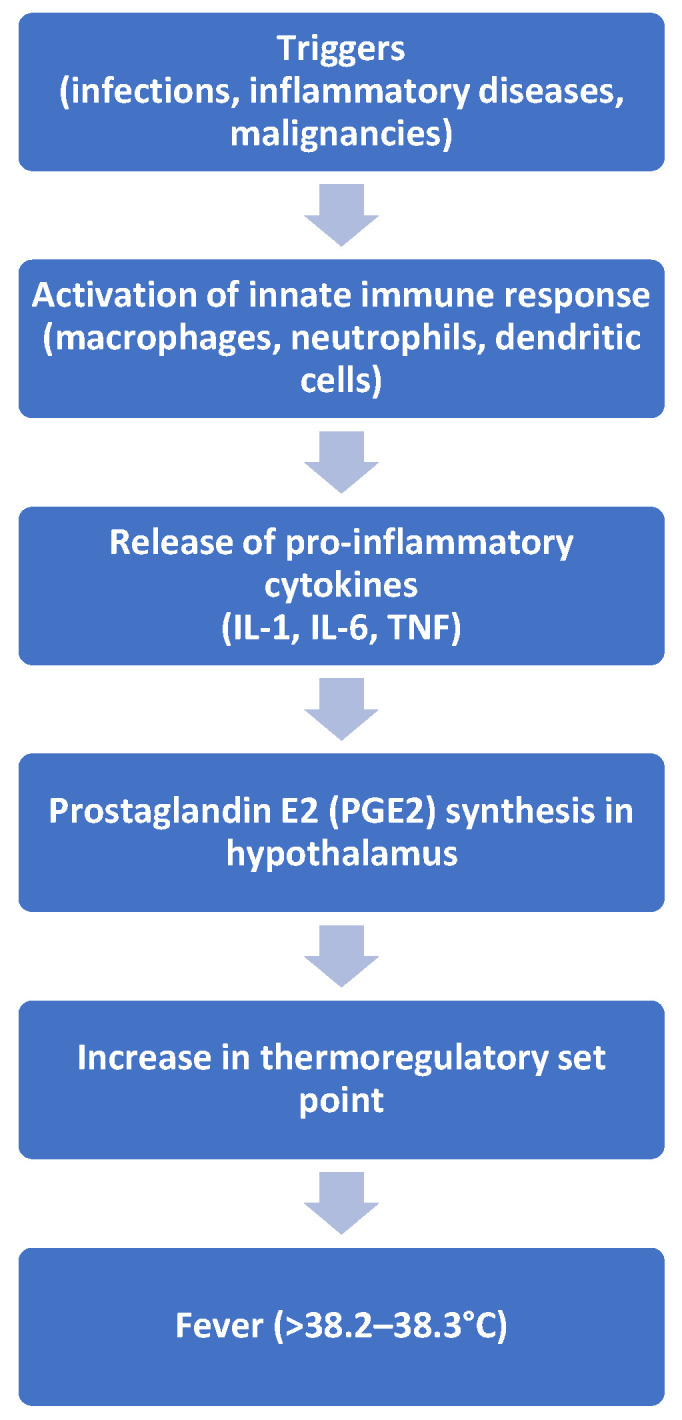
Pathophysiology of fever of unknown origin (FUO) (created by the authors). Gastrointestinal and systemic triggers activate innate immune pathways, leading to cytokine release (IL-1, IL-6, and TNF), stimulation of prostaglandin E_2_ synthesis in the hypothalamus, elevation of the thermoregulatory set point, and development of fever. Additional mechanisms include GALT activation, intestinal dysbiosis, microbial translocation, and NETosi.

The pathophysiological mechanisms underlying FUO are summarized in Figure 1.

## 3. Gastroenterological Causes of FUO

Gastroenterological sources of fever of unknown origin constitute an important subset of diagnoses within the infectious, neoplastic, and autoimmune inflammatory categories of the gastrointestinal tract. Inflammatory bowel diseases (Crohn’s disease and ulcerative colitis) are listed among the most common non-infectious inflammatory conditions associated with FUO, alongside systemic autoimmune disorders and giant cell arteritis [7]. It is estimated that this group accounts for 10–30% of FUO cases [11].

Gastrointestinal infections also play a substantial role in the epidemiology of FUO. Infectious etiologies represent the most prevalent category of FUO and account for approximately 50% of all diagnoses [17,30]. In Europe, their contribution is lower yet remains significant, encompassing bacterial infections such as typhoid fever, salmonellosis, and yersiniosis, as well as parasitic diseases, including visceral leishmaniasis [7,31]. Other parasitic infections implicated in FUO include amebiasis, giardiasis, and toxocariasis [32]. In pediatric populations, the spectrum of infectious causes is age-dependent: bacterial infections are more common in infancy, whereas viral infections predominate in preschool and school-aged children [16]. The spectrum of gastrointestinal causes of FUO also includes rarer entities which, due to their nonspecific clinical course, may remain undiagnosed for a long time. This group includes Whipple’s disease, chronic active hepatitis, and granulomatous peritonitis, as well as more deeply located intra-abdominal abscesses, including retroperitoneal, subphrenic, and psoas abscesses [32]. Particular emphasis should be placed on occult infectious foci located within the abdominal cavity, which in the course of FUO may remain clinically paucisymptomatic despite their significant etiological relevance. In a Turkish analysis, intra-abdominal abscesses represented the second most common cause of FUO within the group of infectious diseases, and liver abscesses were the most frequent form of focal infection [13].

Also, extrapulmonary tuberculosis involving the abdominal cavity may present as isolated prolonged fever without accompanying features of pulmonary disease. Such a clinical presentation increases the risk of delayed diagnosis, especially when local symptoms are sparse or nonspecific. Therefore, in cases with concomitant ascites or other findings suggestive of peritoneal involvement, peritoneal tuberculosis should be considered a possible source of fever of unknown origin [7].

Among the non-infectious inflammatory diseases associated with FUO, hepatic and biliary disorders are frequently noted [7]. This group includes both hepatic abscesses and infections with hepatotropic viruses, although the latter constitute only a marginal proportion of FUO cases [12]. Chronic inflammatory processes capable of causing prolonged fever may also involve viral hepatitis—particularly hepatitis B virus (HBV) infection [17]—as well as autoimmune cholangitis and granulomatous hepatitis [32]. Pancreatic diseases, including acute and chronic pancreatitis, may likewise lead to FUO due to the organ’s capacity to elicit a systemic inflammatory response [15,32].

Gastrointestinal malignancies account for 20–30% of FUO diagnoses [11], with colorectal and gastric cancers being the most frequently implicated [7]. In a Chinese cohort of more than 16,000 patients, gastrointestinal tumors represented 1.1% of neoplastic causes of FUO, a considerably lower frequency compared with lymphomas and other hematologic malignancies [3,12]. In a multicenter study spanning 21 countries, malignancies accounted for 11% of FUO etiologies [17]. Turkish studies have similarly reported FUO associated with lymphomas and gastrointestinal cancers [13], and neuroendocrine tumors (NETs) also play a relevant role [17]. Furthermore, a Danish registry-based study demonstrated a threefold increase in the risk of lower gastrointestinal malignancies within 12 months following an FUO diagnosis [33]. The relationship between FUO and gastrointestinal malignancies also has a temporal dimension, as fever may precede the clinical manifestation of the neoplastic process before a definitive diagnosis is established. It has been shown that the increased number of cancer diagnoses following FUO is greatest during the first months of follow-up, suggesting that in some patients fever represents an early manifestation of an already existing but initially unrecognized disease [33]. The spectrum of gastroenterological disorders associated with fever of unknown origin is broad and encompasses inflammatory, infectious, autoimmune, and neoplastic conditions; the most important entities are summarized in Table 1.

## 4. The Diagnosis of Gastroenterological Causes of FUO

FUO as a consequence of its various etiologies can be a diagnostic challenge for clinicians [1]. The minimum diagnostic tests that ought to be carried out before qualifying a fever as FUO are not broadly adopted [34]. A detailed medical history must be obtained to discover potential diagnostic clues. Drug fever or factitious fever must be excluded [4].

Current FUO criteria are based on Delphi-generated consensus. These criteria include fever of ≥38.3 °C measured on ≥3 occasions and lasting ≥ 3 weeks without explanation following a set of minimal standard diagnostic tests in an immunocompetent patient [7,35]. In the Delphi-generated consensus, panelists were in favor of lowering the threshold from 38.3 °C to 38°C, but it did not reach a consensus. According to the consensus recommendation, the minimum obligatory tests include (Table 2) complete blood count (CBC), metabolic panel with calcium and liver tests, erythrocyte sedimentation rate (ESR), C-reactive protein (CRP), ferritin, thyroid-stimulating hormone (TSH), rheumatoid factor (RF), antineutrophil cytoplasmic antibodies (ANCAs), antinuclear antibodies (ANAs), human immunodeficiency virus (HIV) 1/2 serology, urinalysis with microscopy, Mycobacterium tuberculosis skin test or whole blood interferon-γ release assay (IGRA), 3 sets of blood cultures, and imaging studies. Imaging studies include both abdominal ultrasonography and posteroanterior lateral view chest plain radiography or chest/abdominal/pelvic computed tomography (CT). In the Delphi-generated consensus, the panel recommends the use of 2-deoxy-2-[18F] fluoro-D-glucose positron emission tomography (^18^FDG-PET)/CT, when available, for diagnosing a patient meeting FUO criteria after minimal diagnostic tests, but it should not be combined into the diagnostic criteria of FUO [35].

When liver function tests are abnormal, hepatitis serology should be performed [36,37]. Immunocompromised patients are patients with neutropenia for ≥one week during the three months preceding the fever, HIV infection, hypogammaglobulinemia, or the administration of 10 mg of prednisone or the equivalent for at least two weeks during the three months preceding the fever [4].

When available, FDG-PET or CT may be useful in diagnosing FUO. FDG-PET can detect anatomic localization of infection, inflammation and neoplastic processes [36]. FDG-PET/CT is especially useful in detecting potential tumors or tumors in problematic locations [12]. Lower than FDG-PET/CT diagnostic yield methods such as labeled leukocyte scintigraphy or gallium scintigraphy may be performed when FDG-PET/CT is unavailable [4]. Invasive tests may be used when necessary due to the clinical picture or the need for histopathological examination. The most frequent invasive tests in FUO are lymph nodes, liver, bone marrow, epididymal nodule and temporal artery biopsies. For gastrointestinal causes of FUO such as Crohn’s disease, biliary tract disease and gastrointestinal tumor endoscopic examination of the upper and lower gastrointestinal tract and retrograde cholangiography should be performed [36]. FUO research should focus on improving the process of reaching an accurate diagnosis and keeping the process efficient by using the minimum number of tests needed [30].

## 5. Therapeutic Management

Therapeutic management in cases of FUO with a potential gastroenterological origin is based on a stepwise assessment of the treatment response and on the continuous verification of diagnostic hypotheses as new clinical data become available [38]. Interventions implemented in the course of FUO must be closely aligned with clinical findings, as a substantial proportion of cases (approximately 45%) resolve spontaneously, and non-targeted treatment may obscure the clinical picture or induce adverse effects [7,16,17,30,37]. This applies particularly to glucocorticoids, which should be administered only after confirming autoimmune disease and excluding malignancy, as they may mask key symptoms and hinder accurate diagnosis [15]. Effective therapy can be achieved only once the underlying disease mechanism responsible for the symptoms is identified [10,11,29]. Current recommendations advise withholding causal treatment until the full diagnostic workup has been completed. Exceptions include high-risk scenarios in which delayed intervention poses an immediate threat to life, for example, neutropenia, severe immunosuppression, or critical illness, where prompt antimicrobial therapy is mandatory [7,11,14,29].

Limiting nonspecific, repeated antibacterial treatment before establishing a definitive diagnosis is also important, as it may not only delay the initiation of targeted therapy but also modify the clinical presentation and increase the risk of organ complications. In an analysis of patients with brucellosis hospitalized with a clinical picture of FUO, these patients were shown to have a very high rate of misdiagnosis, and an irregular fever pattern was associated, among other factors, with prior use of antipyretics and antibiotics before hospital admission [6]. In contrast, among patients with HIV/AIDS, most etiologies of FUO were potentially treatable, and the authors emphasized the need to select diagnostic tests according to the patient’s immunological status [3].

In cases suggesting an immunological or autoinflammatory origin of fever, particularly when accompanied by cutaneous manifestations or markedly elevated ferritin levels, glucocorticoids are employed [38,39]. In several situations, including suspected autoinflammatory disorders or drug hypersensitivity syndromes, the introduction of additional immunosuppressive agents, such as cyclosporine, has proven necessary [38].

Management of FUO is primarily supportive, with treatment intensity determined by symptom dynamics and the risk of complications. In cases of persistent fever, broad-spectrum antibiotics or antifungal agents may be required to minimize the risk of progression of an unidentified infectious process [5,40]. Once pathogens are identified, targeted antimicrobial therapy should be initiated [21]. Continuous monitoring of clinical and biochemical parameters is essential, including evaluation of temperature patterns, trends in inflammatory markers, and organ function [5]. In cases where the clinical presentation suggests that the gastrointestinal tract contributes to the persistence of the febrile process, supportive management should also include early assessment of nutritional status and avoidance of unjustified prolongation of fasting, as the absence of enteral stimulation leads to impairment of GALT function, decreased sIgA secretion, weakening of the epithelial barrier, and increased bacterial translocation. Nevertheless, symptomatic therapy remains an important component of FUO management. Paracetamol is the most commonly used antipyretic, a choice justified by its effectiveness and by its relatively limited impact on the diagnostic process compared with anti-inflammatory agents [41].

## 6. Final Message

FUO due to its numerous causes poses a diagnostic challenge to clinicians [1]. Gastroenterological causes are an important part of FUO diagnosis as they represent 10–30% of FUO cases, with IBD being one of the main cause of NIID [7,11]. With no minimum diagnostic test being adopted for diagnosing a fever as FUO, future research should focus on enhancing the diagnosis process by minimizing the number of test needed [30,34]. The aim of this review was to synthesize current research on gastroenterological causes of FUO and highlight the importance of further research on FUO to facilitate the process of reaching the accurate diagnosis.

## Figures and Tables

**Table 1:** Overview of gastroenterological causes of fever of unknown origin (FUO).

Category	Examples
Inflammatory bowel diseases	Crohn’s disease, ulcerative colitis
Intra-abdominal infections	Liver abscess, subphrenic abscess, psoas abscess, and retroperitoneal abscess
Hepatobiliary diseases	Viral hepatitis (HBV), autoimmune cholangitis, and granulomatous hepatitis
Pancreatic diseases	Acute pancreatitis, chronic pancreatitis
Gastrointestinal infections	Salmonellosis, typhoid fever, yersiniosis, amebiasis, giardiasis, toxocariasis, and visceral leishmaniasis
Granulomatous and rare diseases	Whipple’s disease, peritoneal tuberculosis
Malignancies	Colorectal cancer, gastric cancer, and neuroendocrine tumors

**Table 2:** The minimum obligatory diagnostic tests according to the Delphi-generated consensus.

Laboratory Tests	Microbiology Tests	Imaging Studies
Complete blood count (CBC), erythrocyte sedimentation rate (ESR), C-reactive protein (CRP), Calcium, Liver tests, Ferritin, thyroid-stimulating hormone (TSH), rheumatoid factor (RF), antineutrophil cytoplasmic antibodies (ANCAs), antinuclear antibodies (ANAs)	HIV 1/2 serology, urinalysis with microscopy, Mycobacterium tuberculosis skin test or IGRA, and blood cultures (3 sets)	Both abdominal ultrasonography and posterioanterior lateral view chest plain radiography or chest/abdominal/pelvic CT

## Data Availability

No new data where created or analyzed in this study.
